# Toll-Like Receptor 4 Is an Early and Sensitive Biomarker to Detect Acute Kidney Injury after Surgery for Type A Aortic Dissection

**DOI:** 10.31083/j.rcm2311363

**Published:** 2022-10-25

**Authors:** Jingfang Xu, Zhigang Wang, Qingyan Zhang, Dongjin Wang, Chunming Jiang, Hengjin Wang

**Affiliations:** ^1^Department of Nephrology, Nanjing Drum Tower Hospital Clinical College of Nanjing University of Chinese Medicine, 210008 Nanjing, Jiangsu, China; ^2^Department of Cardio-thoracic Surgery, Affiliated Drum Tower Hospital, Medical School of Nanjing University, 210008 Nanjing, Jiangsu, China; ^3^Department of Nephrology, Affiliated Drum Tower Hospital, Medical School of Nanjing University, 210008 Nanjing, Jiangsu, China

**Keywords:** toll-like receptor 4, acute kidney injury, aortic dissection, 30-day mortality, risk factor

## Abstract

**Background:**

Acute kidney injury (AKI) is a relatively common complication after surgery for type A acute aortic dissection (ATAAD) and is associated with a poor prognosis. Preclinical models suggest that toll-like receptor 4 (TLR4) may participate in the pathogenesis of AKI. However, the correlation of serum TLR4 and post-operative AKI has not been studied in ATAAD patients. This study aimed to explore the possibility of using serum TLR4 levels to predict AKI and 30-day mortality in patients undergoing ATAAD surgery.

**Methods:**

A prospective, single-center cohort study was conducted and enrolled a total of 64 patients undergoing ATAAD surgery. The level of serum TLR4 was measured and compared before and within 24 hours after the completion of surgery.

**Results:**

Thirty-five (54.7%) patients developed AKI, including 7 (10.9%) diagnosed with severe AKI (Kidney Disease Improving Global Outcomes (KDIGO) stage 3). TLR4 levels at 0-hour,1-hour, 3-hour, and 6-hour after intensive care unit (ICU) admission were significantly different between patients with or without AKI. Further analysis showed that the difference was most significant at 0-hour after ICU admission which corresponded to an area under the curve (AUC) of 0.886 (95% confidence interval (CI), 0.800 to 0.973). For severe AKI, the AUC of TLR4 was the highest with 0.923 (0.852 to 0.995) at 1-hour after ICU admission. TLR4 levels before surgery and at 0-hour, 1-hour, as well as 3-hour after ICU admission were significantly different between survivors and non-survivors. Furthermore, we found that the serum level of TLR4 upon ICU admission could be used to predict the 30-day mortality with AUC of 0.805 (0.648 to 0.962).

**Conclusions:**

Serum TLR4 levels can be used as a biomarker to predict the occurrence of AKI and 30-day mortality in patients undergoing ATAAD surgery.

**Clinical Trial Registration Number:**

ChiCTR2200057197.

## 1. Introduction

Toll-like receptor 4 (TLR4) is an important pattern recognition receptor mainly expressed on renal tubular epithelial cells and vascular endothelial cells that mediates the nuclear factor (NF)-κB inflammatory cascade and participates in the pathogenesis of acute kidney injury (AKI) [1]. The expression of renal TLR4 remains low under physiological conditions and increases upon renal injury. TLR4 activation induces the expression of NF-κB-dependent proinflammatory cytokines such as tumor necrosis factor-α, interleukin-6, and interleukin-1β [2]. These cytokines can further induce tubular epithelial cell necrosis and renal tubular atrophy [3]. The correlation between TLR4 and AKI has been reported in several preclinical models [4]. In sepsis-induced AKI, increased expression of renal TLR4 was found in proximal and distal tubules as well as in peritubular and glomerular capillaries [5]. A previous study showed that TLR4 expression was upregulated in a cisplatin-mediated AKI model [6], cyclosporin induced nephrotoxicity [7], lupus nephritis [8], unilateral ureter obstruction [9], diabetic nephropathy [10], and rhabdomyolysis-induced AKI [11]. However, the expression of serum TLR4 in patients who developed AKI following surgery has not been well studied.

Acute type A aortic dissection (ATAAD) is a life-threatening condition that is associated with high mortality, not only due to the disease itself, but also due to surgical related major complications [12, 13, 14, 15]. AKI is a relatively common and severe complication of ATAAD surgery and is often associated with poor prognosis. AKI may develop in up to 20% to 67% of all patients who undergo ATAAD surgery [16, 17], which can decrease long-term survival and quality of life, even though the dissection has been successfully repaired [17]. Therefore, early identification of patients with a high risk for developing AKI after ATAAD surgery would help to improve their immediate and long-term prognosis.

Conventional markers such as serum creatine (sCr) level and urinary output can be affected by many factors during the postoperative period, and therefore have limited value in the early diagnosis of AKI [18, 19]. Novel biomarkers, such as cystatin C, have showed less sensitivity for AKI than sCr levels [20]. Comprehensive measurements such as renal resistive index, have been proposed to be a useful tool to predict AKI after ATAAD surgery but might be influenced by heart rate and mean arterial pressure [21].

Therefore, we conducted a prospective study to evaluate the value of TLR4 levels at different time points to determine the early diagnosis of AKI in ATAAD patients who underwent reparative surgery and their association with 30-day mortality.

## 2. Materials and Methods

### 2.1 Study Population

**Fig. 1.**
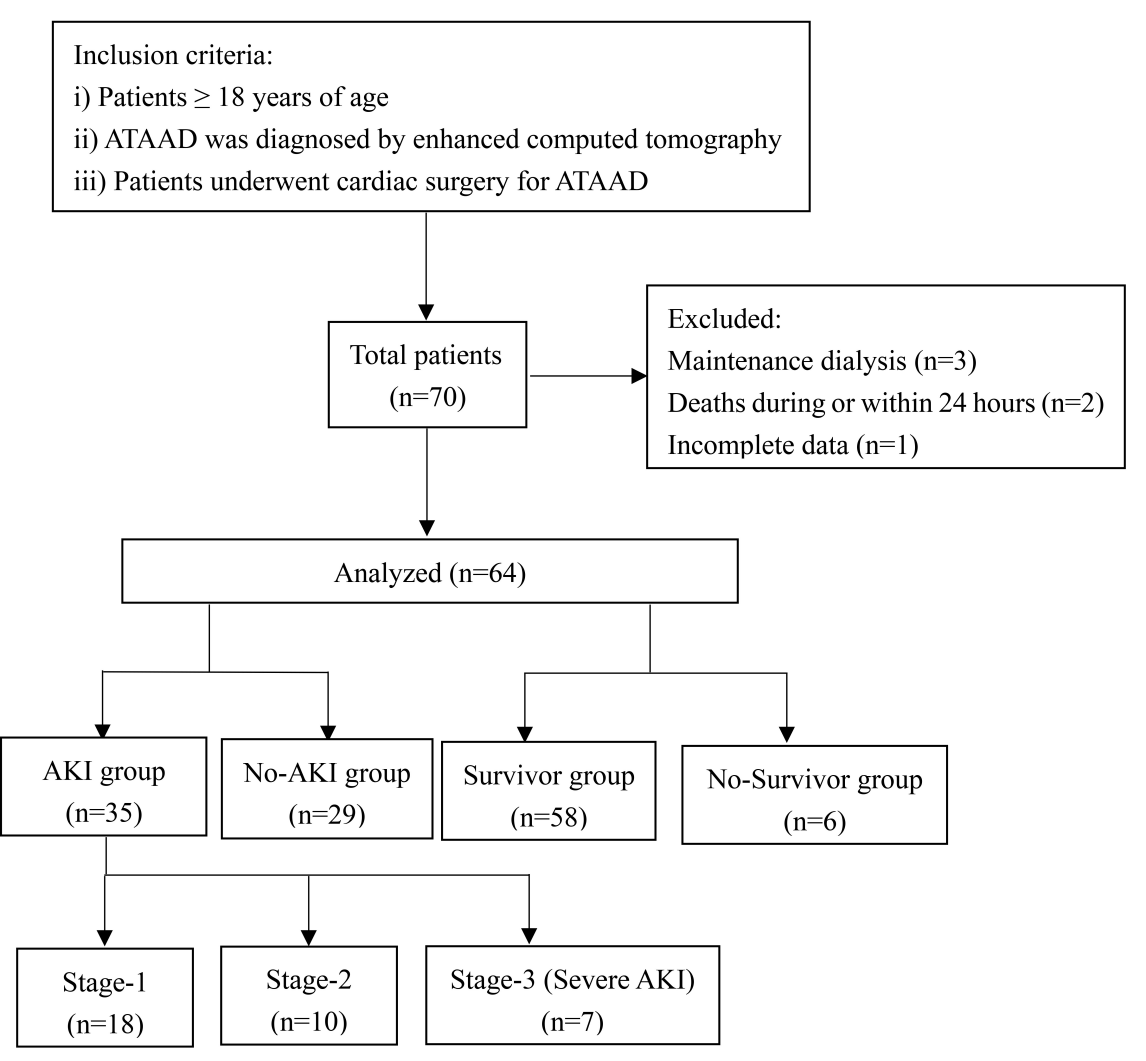
**The patient selection process**.

70 adult patients who were diagnosed with ATAAD by enhanced computed tomography (CT) and received surgery within 14 days of disease onset were enrolled in this prospective, observational single-center study. The study was conducted between December 29 2021 and April 25 2022. As shown in Fig. 1, patients on renal replacement therapy (RRT) before surgery (n = 3) and who died during or within 24 hours after surgery (n = 2) were excluded from the final analysis because of the difficulty in measuring the progression of renal dysfunction. In addition, 1 patient was excluded due to incomplete data. All patients received standard of care and were transferred to the intensive care unit (ICU) after the completion of surgery. Prompt resuscitation of the circulation with fluids, vasopressors and inotropes was applied after patients were diagnosed with AKI.

### 2.2 Definition of AKI

Postoperative AKI was diagnosed according to the Kidney Disease Improving Global Outcomes (KDIGO) criteria [22] by measuring the change of sCr levels and urine output. The severity of AKI was determined according to the KDIGO guidelines as follows: stage-1: increase of sCr by ≥0.3 mg/dL (≥26.5 μmol/L) within 48 hours or increase by 1.5~1.9 times compared to baseline within 7 days; AKI stage-2: increase of sCr by 2.0~2.9 times compared to baseline; AKI stage-3: increase of sCr by 3 times compared to baseline or an increase of at least 354 μmol/L or when RRT is required.

### 2.3 Data Collection

Patient information including demographic characteristics, medical histories, physical examination results, laboratory tests, imaging findings, treatments and outcomes were collected. All enhanced CT images were independently evaluated by 2 experienced radiologists and disagreements were resolved by further consultation with a third radiologist.

### 2.4 Sample Collection and Biomarker Measurement

2 mL of venous blood was collected from each patient before surgery and at 0-hour, 1-hour, 3-hour, 6-hour, 12-hour, and 24-hour after ICU admission following surgery. Blood samples were centrifuged at 1500 × g for 15 minutes before the supernatant was collected and stored at –80 °C for further analysis. The level of TLR4 was determined by a human TLR4 ELISA kit (RK02399, ABclonal, Nanjing, China), according to the manufacturer’s instruction. The entire ELISA procedure was typically completed in 4 hours and each test cost about 16 RMB.

### 2.5 Outcome Variables

The primary outcome was the difference in serum TLR4 levels between patients with and without postoperative AKI at different timepoints. Secondary outcomes included the correlation between changes of serum TLR4 levels at different timepoints and stage-3 AKI as well as 30-day mortality.

### 2.6 Surgical Procedures

The operation procedure performed in this study was described in our previous study [16]. Briefly, cardiopulmonary bypass (CPB) was established by cannulation of femoral artery or right axillary artery with right atrium. Cardiac arrest was accomplished with cold blood cardioplegia (4:1 blood:crystalloid ratio) which was infused by both anterograde and retrograde infusion method. Circulation arrest was initiated when cooling reached its target rectal temperature of 22 °C, and the temperature maintained 18–22 °C during the circulation arrest period. Total arch replacement plus frozen elephant trunk method was selected when major dissection teared around aortic arch or proximal descending aorta. To prevent postoperative AKI, the mean arterial pressure was maintained between 55 and 75 mmHg and the urine output was recorded per hour during the surgery.

### 2.7 Statistical Analysis

SPSS 26.0 software (IBM Corp, Armonk, NY, USA) was used for all statistical analyses. Quantitative data was presented as mean ± standard deviation (for normally distributed data) or median with interquartile range (IQR) (for nonnormally distributed data). Qualitative data was expressed as numbers (percentages). The χ^2^ test or Fisher’s exact test was used to compare qualitative data. The* t*-test was applied for normally distributed continuous variables whereas the Mann-Whitney *U*-test was used for non-normally distributed variables.

According to retrospective study in our center, the incidence of postoperative AKI in ATAAD patients was 50.4% [16]. Therefore, we assumed that the incidence of AKI during the study period was 50.0%. The sample size calculation showed that a sample of 28 from the AKI group and 28 from the non-AKI group would achieve 80% power to detect a difference of 0.20 between the area under the receiver operating characteristic (ROC) curve (AUC) under the null hypothesis of 0.80 and an AUC under the alternative hypothesis of 0.7000 using a two-sided z-test at a significance level of 0.05.

To identify independent predictors of AKI and 30-day mortality, multivariate logistic regression analyses were performed including variables with *p*-value < 0.2 identified by univariable analyses. Receiver operating characteristic (ROC) curves were constructed, and the AUC was determined to assess the discriminant ability of serum TLR4 expression levels at different time points in predicting AKI. The optimal cut-off point was determined by Youden’s index (J = sensitivity + specificity – 1). Odds ratios (OR) were reported with 95% confidence interval (CI). For all analyses, a two-tailed *p*-value < 0.05 indicated statistical significance.

## 3. Results

A total of 64 patients, including 60 with DeBakey type I and 4 with DeBakey type II aortic dissections, were included in the analysis (Fig. 1). Among these 64 patients, 35 patients (54.7%) developed AKI, including 18 patients (51.4%) characterized with KDIGO stage-1, 10 (28.6%) with stage-2, and 7 (20.0%) with stage-3. In addition, 5 patients (7.8%) required RRT. KDIGO AKI stages and criteria are summarized in Table 1. The median age was 57.5 years (range, 35 to 83 years) and 47 (73.4%) were males. The average body mass index was 25.9 ± 3.7 kg/$m^{2}$. The most common comorbidities were hypertension (82.8%), cardiovascular disease (21.9%), cerebrovascular disease (14.1%), diabetes (3.1%), and 6.3% of all patients had previous cardiac operations. In the enhanced CT studies, renal artery involvement was identified in 35 patients (54.7%) (Table 2). Total arch replacement was performed in 28 patients (43.8%), concomitant coronary artery bypass grafting was performed in 2 patients (3.1%), and a concomitant Bentall procedure was performed in 5 patients (7.8%). The mean duration for CPB, aortic cross-clamping and operation time were 80.0 ± 48.4 minutes, 131.0 ± 39.9 minutes, and 6.6 ± 1.7 hours respectively. Deep hypothermic circulatory arrest was used in all patients for a mean duration of 26.0 ± 9.1 minutes (Table 3). 6 patients died within 30 days after surgery (9.7%) (Table 4).

**Table 1.** **AKI according to KDIGO criteria**.

Patients	sCr criterion only	UO criterion only	sCr and UO criteria	RRT and sCr criteria	RRT and UO criteria	RRT, sCr, and UO criteria	All criteria AKI, n = 35 (% of AKI patients)
KDIGO stage 1	10	4	4	0	0	0	18 (51.4)
KDIGO stage 2	5	2	3	0	0	0	10 (28.6)
KDIGO stage 3	2	0	0	0	1	4	7 (20.0)
Total amount (% of patients with AKI)	17 (48.6)	6 (17.1)	7 (20)	0 (0)	1 (2.9)	4 (11.4)	

sCr, serum creatinine; UO, urine output; RRT, renal replacement therapy; AKI, acute kidney injury; KDIGO, Kidney Disease Improving Global Outcomes.

**Table 2.** **Comparison of preoperative variables**.

Variables	Total (n = 64)	AKI (n = 35)	Non-AKI (n = 29)	*p* value
DeBakey type I (%)	60 (93.8)	34 (97.1)	26 (89.7)	0.321
Time from onset to surgery (hour)	22.8 (12.5, 33.4)	17.6 (8.7, 29.8)	24.8 (14.2, 42.1)	0.031
Presenting variables				
Chest pain (%)	58 (90.6)	31 (88.6)	27 (93.1)	0.681
Back pain (%)	39 (60.9)	20 (57.1)	19 (65.5)	0.494
Abdominal pain (%)	11 (17.2)	7 (20.0)	4 (13.8)	0.741
Vomiting (%)	10 (15.6)	6 (17.1)	4 (13.8)	1.000
Demographic data				
Age (year)	57.5 (46.0, 68.8)	54.0 (43.0, 66.0)	59.0 (48.0, 69.0)	0.202
Male (%)	47 (73.4)	28 (80.0)	19 (65.5)	0.192
BMI (kg/$m^{2}$)	25.9 ± 3.7	26.5 ± 3.9	25.2 ± 3.4	0.167
Medical history				
Hypertension (%)	53 (82.8)	30 (85.7)	23 (79.3)	0.526
Diabetes mellitus (%)	2 (3.1)	2 (5.7)	0 (0)	0.497
Previous cardiovascular disease (%)	14 (21.9)	7 (20.0)	7 (24.1)	0.690
Cerebrovascular disease (%)	9 (14.1)	5 (14.3)	4 (13.8)	1.000
Smoking (%)	16 (25.0)	12 (34.3)	4 (13.8)	0.059
Drinking (%)	10 (15.6)	5 (14.3)	5 (17.2)	1.000
Previous cardiac operation (%)	4 (6.3)	3 (8.6)	1 (3.4)	0.620
PCI (%)	1 (1.6)	1 (2.9)	0 (0)	1.000
TEVAR (%)	3 (4.7)	2 (5.7)	1 (3.4)	1.000
Limb ischemia (%)	11 (17.2)	8 (22.9)	3 (10.3)	0.319
Cerebral ischemia (%)	15 (23.4)	10 (28.6)	5 (17.2)	0.287
Coronary ischemia (%)	4 (6.3)	4 (11.4)	0 (0)	0.120
Involving renal artery (%)	35 (54.7)	26 (74.3)	9 (31.0)	0.001
Hypotension (%)	6 (9.4)	3 (8.6)	3 (10.3)	1.000
Pericardial tamponade (%)	29 (45.3)	18 (51.4)	11 (37.9)	0.280

BMI, body mass index; PCI, percutaneous coronary intervention; TEVAR, thoracic endovascular aortic repair; AKI, acute kidney injury.

**Table 3.** **Comparison of operative variables**.

Variables	Total (n = 64)	AKI (n = 35)	Non-AKI (n = 29)	*p* value
Intro-operative variables				
TAR + FET (%)	28 (43.8)	15 (42.9)	13 (44.8)	0.874
Concomitant CABG (%)	2 (3.1)	2 (5.7)	0 (0)	0.497
Concomitant MVP (%)	2 (3.1)	1 (2.9)	1 (3.4)	1.000
Concomitant AVP (%)	2 (3.1)	1 (2.9)	1 (3.4)	1.000
Bentall (%)	5 (7.8)	3 (8.6)	2 (6.9)	1.000
CPB duration (minute)	180.0 ± 48.4	196.7 ± 56.3	160.3 ± 26.8	0.002
Aortic cross-clamp time (minute)	131.0 ± 39.9	142.5±48.1	117.6± 21.2	0.009
DHCA time (minute)	26.0 ± 9.1	27.2 ± 10.8	24.6 ± 6.6	0.253
Operation time (hour)	6.6 ± 1.7	7.3 ± 1.7	5.7 ± 1.1	<0.001
Lowest nasopharyngeal temperature (°C)	23.3 ± 1.2	23.0 ± 1.2	23.6 ± 1.0	0.066
RBC transfusion (mL)	2125.0 (1662.5, 2645.0)	2275.0 (1900.0, 2980.0)	2075.0 (1525.0, 2412.5)	0.051

TAR, total arch replacement; FET, frozen elephant trunk; CABG, coronary artery bypass graft; MVP, mitral valvuloplasty; AVP, aortic valvuloplasty; CPB, cardiopulmonary bypass; DHCA, deep hypothermic circulatory arrest; RBC, red blood cell; AKI, acute kidney injury.

**Table 4.** **Comparison of postoperative variables**.

Variables	Total (n = 64)	AKI (n = 35)	Non-AKI (n = 29)	*p* value
Postoperative complications (%)	32 (50)	26 (74.3)	6 (20.7)	<0.001
Reintubation (%)	5 (7.8)	5 (14.3)	0 (0)	0.034
Tracheotomy (%)	1 (1.6)	1 (2.9)	0 (0)	1.000
Atrial fibrillation (%)	15 (23.4)	11 (31.4)	4 (13.8)	0.097
ARDS (%)	26 (40.6)	24 (68.6)	2 (6.9)	<0.001
Lung infection (%)	19 (29.7)	17 (48.6)	2 (6.9)	<0.001
SWI (%)	2 (3.1)	2 (5.7)	0 (0)	0.497
CRRT (%)	5 (7.8)	5 (14.3)	0 (0)	0.034
Cerebral infarction (%)	7 (10.9)	6 (17.1)	1 (3.4)	0.116
Delirium (%)	9 (14.1)	7 (20.0)	2 (6.9)	0.166
Paraplegia (%)	2 (3.1)	2 (5.7)	0 (0)	0.497
Osteofascial compatament syndrome (%)	1 (1.6)	1 (2.9)	0 (0)	1.000
Use of diuretics (%)	42 (65.6)	30 (85.7)	12 (41.4)	<0.001
Inotropic support (%)	41 (64.1)	27 (77.1)	14 (48.3)	0.017
Inotropic support >24 hours (%)	30 (46.9)	22 (62.9)	8 (27.6)	0.005
Inotropic support >48 hours (%)	23 (35.9)	19 (54.3)	4 (13.8)	0.001
Drainage volume 24 hours after surgery (mL)	475.0 (300.0, 692.5)	540.0 (345.0, 860.0)	410.0 (300.0, 650.0)	0.112
Ventilation time (hour)	20.0 (12.3, 67.0)	46.0 (17.0, 155.0)	14.0 (7.0, 19.5)	<0.001
30-day mortality (%)	6 (9.4)	6 (17.1)	0 (0)	0.028
ICU stay (day)	3.0 (2.0, 6.0)	5.0 (2.0, 11.5)	2.0 (1.0, 3.0)	<0.001
Hospital stay (day)	14.0 (11.0,19.0)	17.0 (13.0, 22.5)	13.0 (10.0, 17.0)	0.005

ARDS, acute respiratory distress syndrome; SWI, sternal wound infection; CRRT, continuous renal replacement therapy; ICU, intensive care unit; AKI, acute kidney injury.

**Table 5.** **Comparison of laboratory tests upon admission**.

Variables	Total (n = 64)	AKI (n = 35)	Non-AKI (n = 29)	*p* value
WBC (${\mathrm{10}}^{9}$/L)	11.2 (8.8, 14.3)	13.2 (9.8, 15.2)	10.2 (7.6, 11.9)	0.012
Haemoglobin (g/L)	126.0 ± 18.5	131.8 ± 19.9	121.8 ± 16.3	0.808
PLT (${\mathrm{10}}^{9}$/L)	163.8 ± 67.9	161.1 ± 42.2	161.5 ± 116.6	0.309
Triglyceride (mmol/L)	1.2 ± 1.1	1.6 ± 1.3	0.7 ± 0.3	0.061
CRP (mg/dL)	10.1 (3.8, 31.1)	8.6 (4.1, 13.7)	10.1 (1.5, 31.1)	0.626
D-dimer (ng/mL)	7.4 (3.3, 21.6)	7.1 (5.0, 65.7)	4.5 (2.26, 21.7)	0.314
Albumin (g/L)	39.0 (35.5, 41.1)	39.8 (35.7, 42.8)	39.2 (38.6, 41.9)	0.451
TnT (ng/mL)	0.016 (0.009, 0.065)	0.017 (0.009, 0.199)	0.015 (0.008, 0.127)	0.009
ALT (U/L)	25.1 (17.0, 42.0)	27.3 (20.9, 48.6)	25.9 (13.1, 88.4)	0.766
Bun (mmol/L)	6.9 ± 2.0	7.4 ± 2.2	6.3 ± 1.5	0.028
sCr (μmoI/L)	88.4 ± 36.8	102.1 ± 48.3	74.0 ± 24.6	0.003
BNP (pg/mL)	106.8 ± 120.5	101.3 ± 110.7	123.1 ± 137.8	0.571
Total bilirubin (mg/dL)	16.1 ± 6.5	14.1 ± 5.4	17.0 ± 6.2	0.132
PT (s)	12.1 (11.3, 13.2)	12.2 (11.4, 13.3)	11.9 (10.8, 12.5)	0.134
APTT (s)	27.3 (25.7, 29.5)	26.7 (25.7, 30.0)	27.4 (24.9, 28.3)	0.405
Fibrinogen (g/L)	2.3 ± 1.4	2.2± 1.2	2.6 ± 1.6	0.220
INR	1.06 (1.00, 1.18)	1.07 (1.01, 1.17)	1.05 (0.95, 1.10)	0.619
Serum lactate (mmol/L)	2.9 ± 1.4	2.9 ± 1.4	2.7 ± 1.4	0.787

WBC, white blood cell; PLT, platelet; CRP, c-reactive protein; TnT, troponin T; ALT, alanine aminotransferase; Bun, blood urea nitrogen; sCr, serum creatinine; BNP, brain natriuretic peptide; PT, prothrombin time; APTT, Activated Partial Thromboplastin Time; INR, international normalized ratio; AKI, acute kidney injury.

Significant differences in the time from disease onset to operation, renal artery involvement, white blood cell, troponin T, blood urea nitrogen, and sCr level were identified between patients with and without AKI (Tables 2,5). Operative parameters including CPB time, aortic cross-clamp time, and operation time were significantly different between the 2 groups (Table 3). The incidence of postoperative complications including reintubation (14.3% vs. 0, *p* = 0.034), acute respiratory distress syndrome (68.6% vs. 6.9%, *p *< 0.001), lung infection (48.6% vs. 6.9%, *p *< 0.001), and the requirement for RRT (14.3% vs. 0, *p* = 0.034) were significantly increased in patients who developed AKI. In addition, patients who developed AKI had longer ventilation times, increased 30-day mortality (17.1% vs. 0, *p* = 0.028), and longer ICU and overall hospitalization stays (Table 4).

Multivariate analysis revealed that increased level of TLR4 at 0-hour upon ICU admission was identified as a risk factor for developing postoperative AKI (OR 3.046, 95% CI 1.435–7.024; *p* = 0.006) and increased 30-day mortality (OR 2.604, 95% CI 1.039–6.002; *p* = 0.016).

**Fig. 2.**
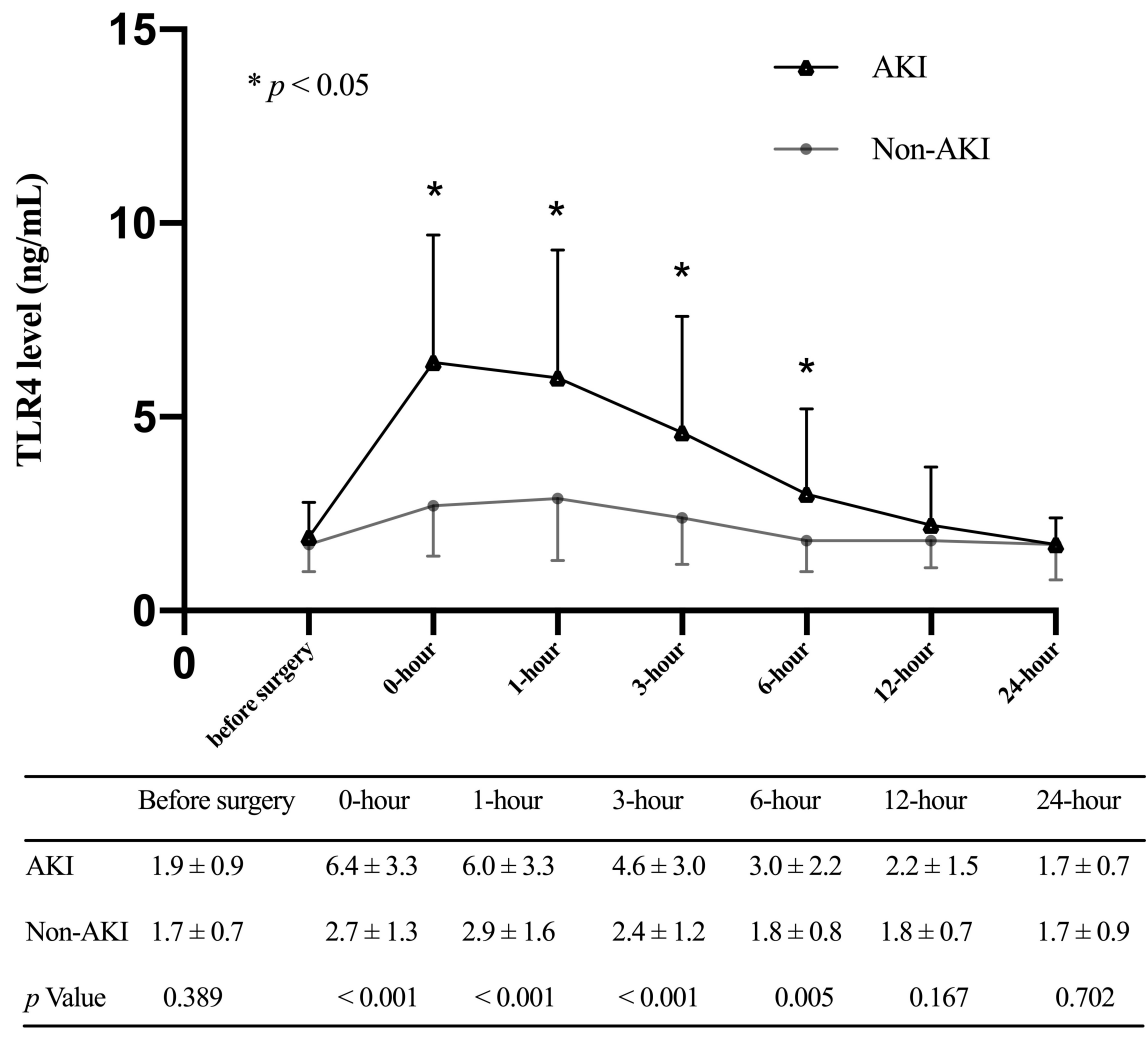
**Toll-like receptor 4 levels (mean and standard deviation) of patients in the AKI group and the non-AKI group at multiple time points**.

**Fig. 3.**
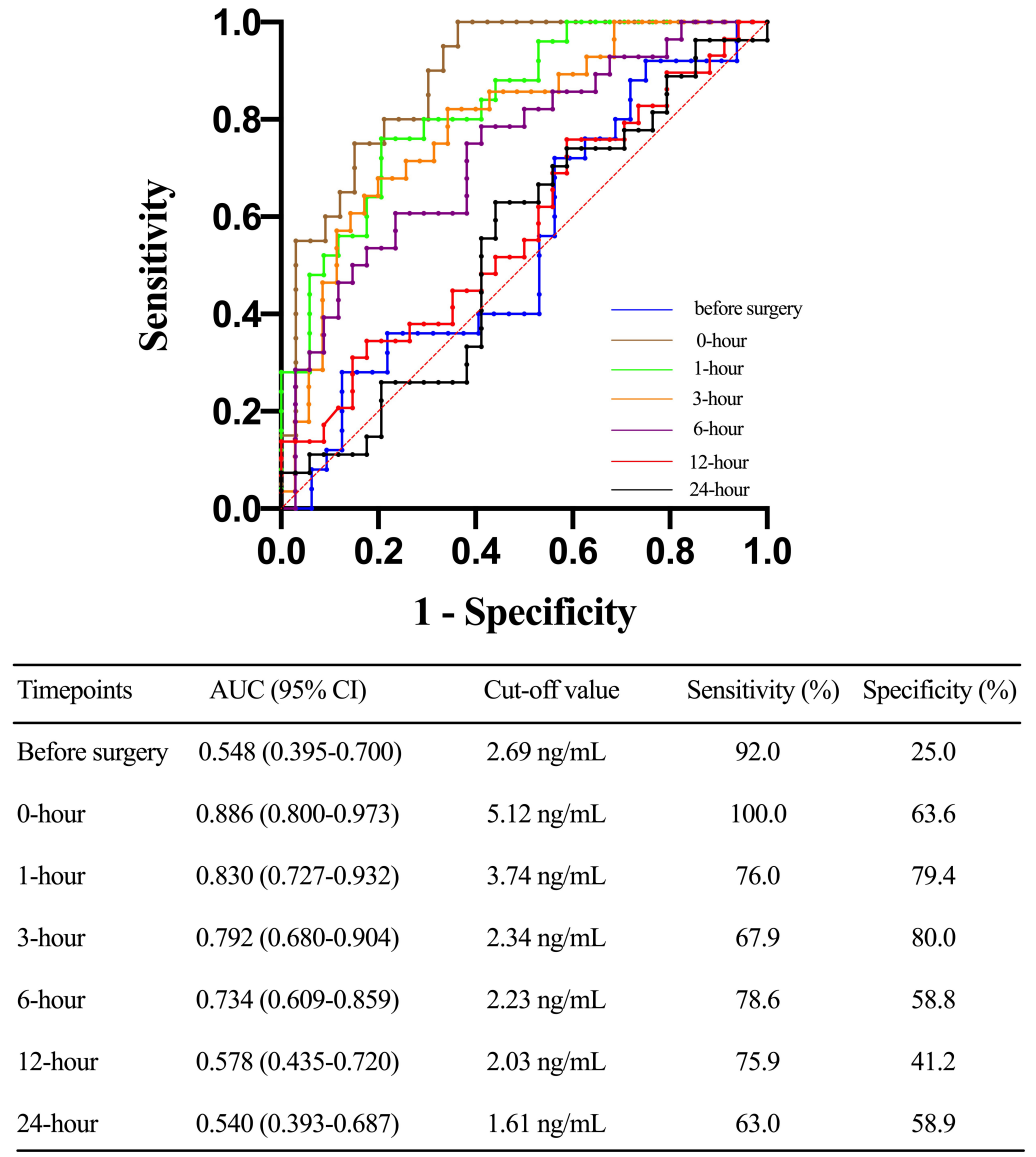
**Receiver operating characteristic (ROC) curves before surgery, 0-hour, 1-hour, 3-hour, 6-hour, 12-hour, and 24-hour toll-like receptor 4 levels after ICU stay in predicting acute kidney injury (AKI) after acute type A aortic dissection (ATAAD) surgery**. AUC, area under the curve; CI, confidence interval.

The mean preoperative TLR4 of all patients was 1.8 ± 0.8 ng/mL, increased steadily, and peaked at 5.0 ± 3.3 ng/mL upon ICU admission, followed by a subsequent downward trend. TLR4 levels at 0-hour, 1-hour, 3-hour, and 6-hour after ICU admission were significantly different between the AKI and the non-AKI groups (Fig. 2). The ROC analysis showed that serum TLR4 concentration at 0-hour after ICU admission was associated with the highest predict value, with an AUC of 0.886 (95% CI, 0.800 to 0.973). In addition, the AUC of serum TLR4 levels at 1-hour, 3-hour, and 6-hour after ICU admission were 0.830 (0.727 to 0.932), 0.792 (0.680 to 0.904), and 0.734 (0.609 to 0.859), respectively (Fig. 3).

**Fig. 4.**
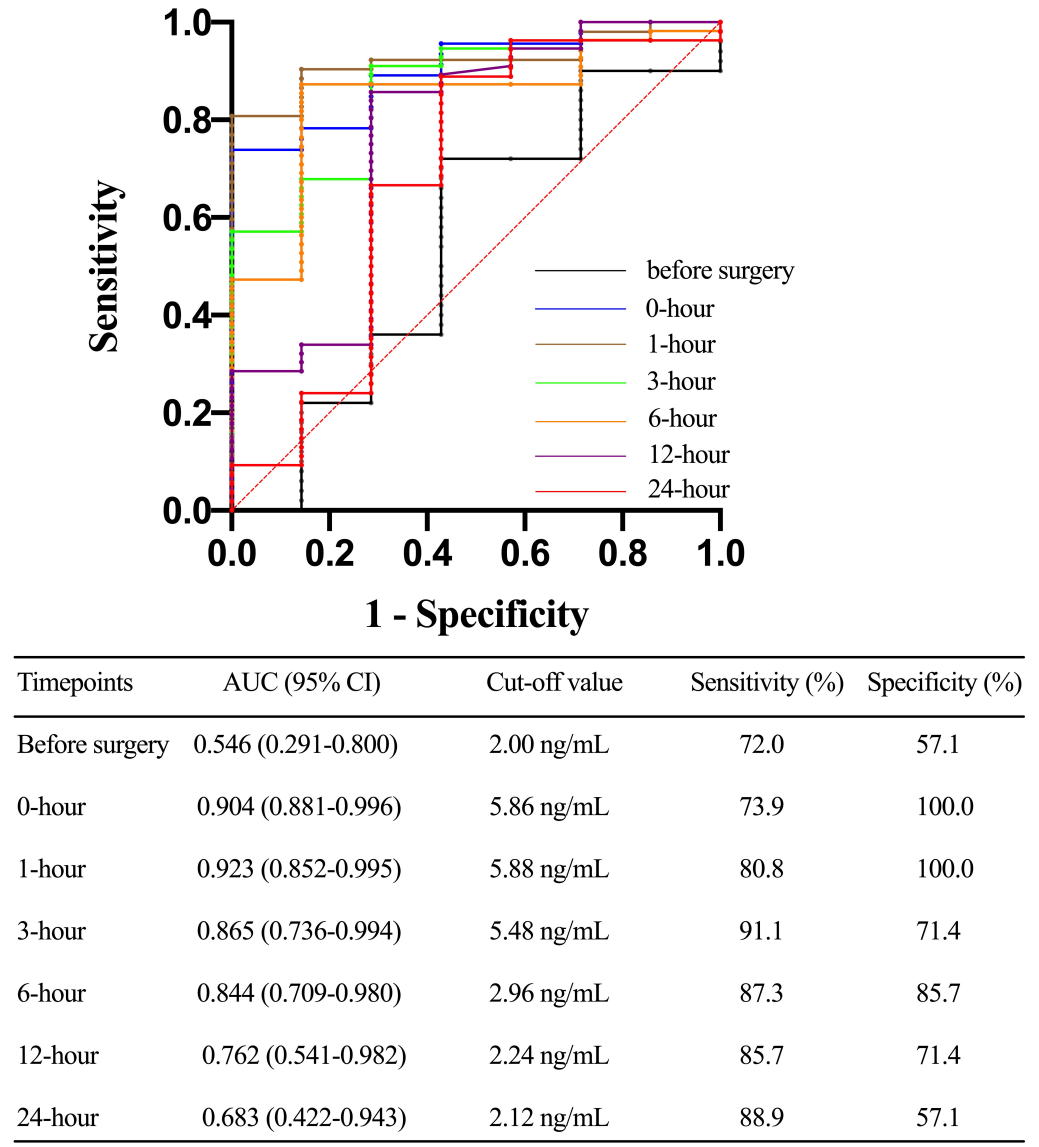
**Receiver operating characteristic (ROC) curve comparing prior to surgery, 0-hour, 1-hour, 3-hour, 6-hour, 12-hour, and 24-hour toll-like receptor 4 levels after ICU stay in predicting acute kidney injury (AKI) (stage 3 AKI) after acute type A aortic dissection (ATAAD) surgery**. AUC, area under the curve; CI, confidence interval.

**Fig. 5.**
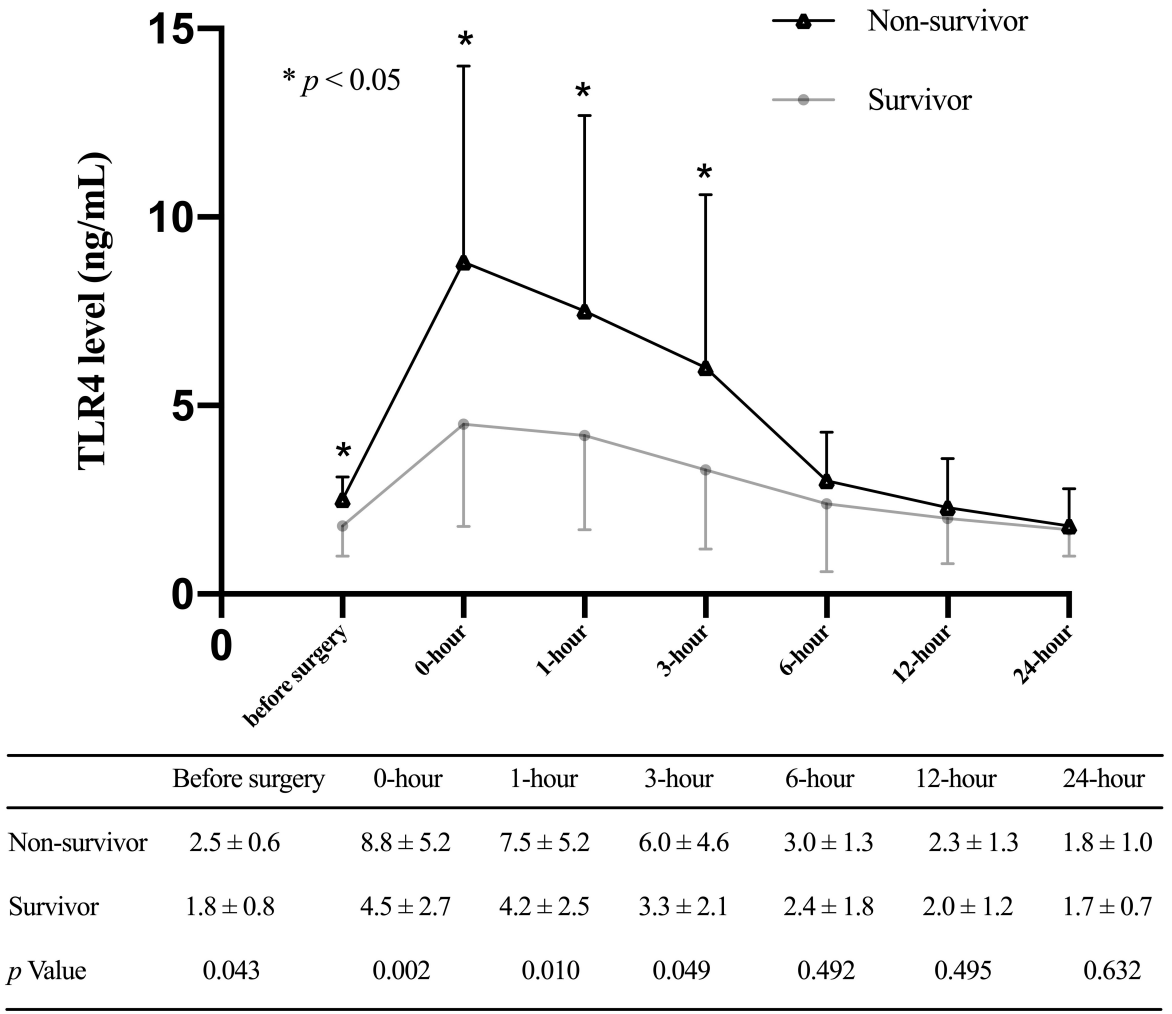
**Toll-like receptor 4 levels (mean and standard deviation) of patients stratified with 30-day mortality at multiple time points**.

**Fig. 6.**
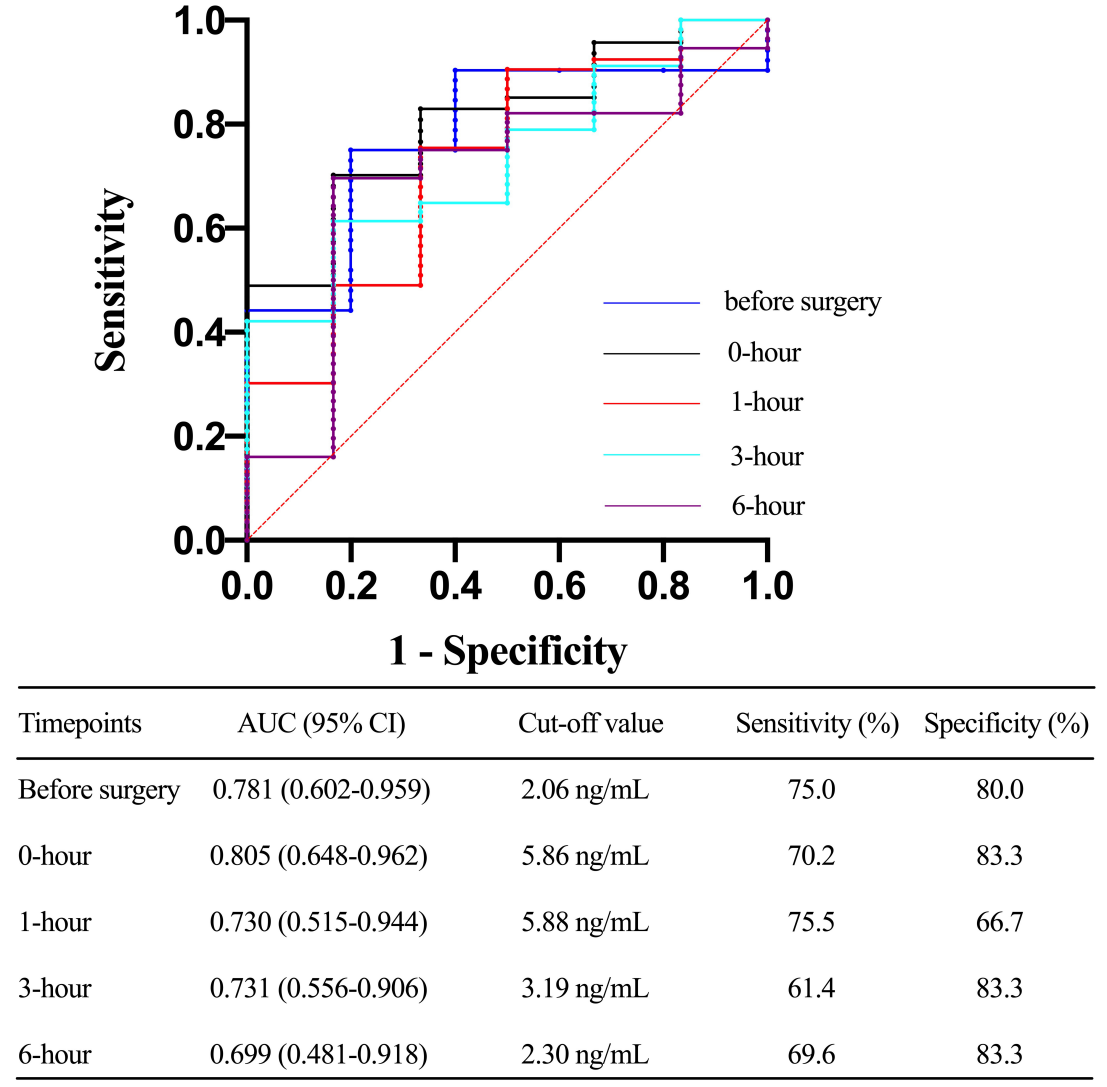
**Receiver operating characteristic (ROC) curve comparing the ability before surgery, 0-hour, 1-hour, 3-hour, 6-hour, 12-hour, and 24-hour toll-like receptor 4 levels after ICU stay in predicting 30-day mortality after acute type A aortic dissection (ATAAD) surgery**. AUC, area under the curve; CI, confidence interval.

The TLR4 concentration at 1-hour after ICU admission could predict the occurrence of severe AKI with an AUC of 0.923 (0.852 to 0.995). The AUC of serum TLR4 levels at 1-hour, 3-hour, and 6-hour after ICU admission to predict severe AKI were 0.904 (0.811 to 0.996), 0.865 (0.736 to 0.994), and 0.844 (0.708 to 0.980), respectively (Fig. 4). TLR4 levels before surgery, 0-hour, 1-hour, and 3-hour after ICU admission were significantly different between survivors and non-survivors within 30 days after surgery (Fig. 5) and could be used to predict 30-day mortality with corresponding AUCs of 0.781 (0.602 to 0.959), 0.805 (0.648 to 0.962), 0.730 (0.515 to 0.944), and 0.731 (0.556 to 0.906), respectively (Fig. 6).

## 4. Discussion

It has been shown in previous studies that the duration of AKI is associated with increased long-term mortality in patients who undergoing surgery for ATAAD [23]. On the other hand, early recovery of renal function after cardiac surgery is associated with improved short- and long-term survival [24]. Therefore, it is important to identify those patients with increased risk for AKI so that appropriate interventions and management can be instituted prior to, during and following surgery. To the best of our knowledge, this is the first study to demonstrate that elevated postoperative TLR4 levels are associated with a statistical increased incidence of AKI and 30-day mortality after adjusting for clinical covariates. The ROC analyses revealed that the AUCs of TLR4 levels at 0-hour after ICU admission to detect AKI, severe AKI, and 30-day mortality were 0.886, 0.904, and 0.805, respectively. Therefore, TLR4 levels after ICU admission might be a novel predictor for developing postoperative AKI in ATAAD patients undergoing surgical repair. Compared to conventional markers, the measurement of TLR4 is convenient and can be analyzed at different times during the postoperative period.

Similar to previous studies, 54.7% of our patients developed AKI following surgery for ATAAD [25]. Other studies reported a higher AKI occurrence of 67%, which might due to different criteria to diagnose AKI [26]. AKI in acute aortic dissection can be characterized into two subtypes: prerenal or intrinsic [19]. Prerenal AKI after ATAAD occurs due to decreased renal perfusion and is reversible. Prompt recognition and rapid restoration of renal perfusion may attenuate or even prevent acute tubular necrosis [27]. The use of RRT when indicated may also improve outcomes [28].

Toll-like receptors (TLRs) are type I membrane-associated glycoproteins that belong to the interleukin-1 receptor super-family [29]. TLRs mainly mediate the function of the innate immune system upon activation with pathogen-associated molecular patterns [30]. It has been known that the innate immune system plays a critical role in initiating the inflammatory cascade that leads to kidney damage [31]. TLRs can also recognize endogenous stress signals or damage-associated molecular patterns such as high mobility group box protein 1 (HMGB1), heat shock proteins and hyaluronan [32] and induce production of inflammatory chemokines and cytokines including interferons (IFNs). It has been demonstrated that extracellular HMGB1 released during an ischemic insult could activate the TLR4 receptor in mice, and participated in the pathogenesis of ischemia-reperfusion induced kidney injury [4]. Hyaluronan is mainly expressed in the inner medulla of the kidney but accumulates in the renal cortex under pathologic conditions such as ischemia-reperfusion injury, diabetic nephropathy, glomerulonephritis and allograft rejection [33, 34]. These studies suggest that ligand mediated TLR4 activation plays an important role in acute kidney injury.

TLR4 is the best characterized TLR in AKI. The expression of TLR4 in the kidney is mainly located in proximal and distal tubular epithelial cells [5, 35, 36, 37]. Ischemia associated renal inflammation upregulates the expression of TLR4 mRNA and protein in the epithelium of the distal convoluted tubule, collecting duct, and loop of Henle [37]. The homodimer of TLR4 is formed following ligand binding and intracellular signaling is transmitted through two major downstream pathways: (1) the MyD88-dependent pathway, which activates early NF-κB and induces cytokine production, and (2) the MyD88-independent TRIF (TIR domain-containing adaptor inducing IFN-β)-dependent pathway, which upregulates the expression of type I IFNs and induces delayed NF-κB activation [38].

Most renal damage in AKI occurs in the tubular epithelial cells. Under pathologic conditions such as ischemia, poisoning and inflammation, renal tubular epithelial cells undergo degeneration, apoptosis, necrosis, and shedding [39]. Both innate and adaptive immune responses are involved in AKI. Except for eliminating endogenous and exogenous antigens, overly robust activation of the immune system leads to excessive production of inflammatory mediators that eventually leads to tissue damage [40]. Consequently, direct or indirect suppression of the inflammatory response has been shown to be able to ameliorate renal damage in AKI models [41].

Our data showed that the serum TLR4 levels were increased after the completion of ATAAD surgery and further increased in patients who developed post-operative AKI. The increase of TLR4 might be due to a secondary inflammatory response. Our study demonstrated an increased incidence of lung infections during the postoperative period in the AKI group. Elevated TLR4 levels might play an important role in this process. A previous study reported the therapeutic effects of TLR 4 agonistic antibodies against lung infections in mice [42]. This phenomenon might offer a new strategy for the treatment of lung infections. Additionally, we noticed that the increase of serum TLR4 levels occurred before sCr. These data indicate that extracorporeal circulation performed during surgery for ATAAD helps to promote the necroptosis in the kidney that results in an elevation of TLR 4 expression which can be used to predict the occurrence of AKI.

Previous studies confirmed that only stage 3 AKI, but not stage 1 or 2, was associated with higher postoperative mortality [43, 44, 45]. However, AKI after cardiac surgery, even in its mild form, was associated with worse short-term outcomes including 30- or 90-day mortality and morbidity, and increases medical costs [46, 47]. Our study showed that elevated TLR4 levels were associated with the occurrence of severe AKI and worse 30-day mortality. Therefore, a TLR4 targeted strategy might be a potential novel therapeutic treatment option to prevent the occurrence of AKI following ATAAD surgery.

Due to the high incidence of developing postoperative AKI after ATAAD surgical repair, patients with elevated TLR4 levels immediately after surgery should be regarded as high-risk populations and potential candidates to receive renal protective treatment. Discovering a more accurate cut-off value of TLR4 in future studies with larger sample sizes would further help to earlier diagnose postoperative AKI in ATAAD and might help to guide a more individualized treatment program.

There are several limitations in the present study. First, the sample size was relatively small and was recruited from a single center. Second, this study was not powered to examine the long-term effects of elevated TLR4 expression. Third, as TLR4 plays a vital role in inflammation and infections, infection data involving other organs systems was missing in the current dataset.

## 5. Conclusions

This study showed that elevated TLR4 levels immediately after ATAAD surgery could predict the occurrence of AKI with good sensitivity and specificity. These results suggest that TLR4 might be considered as a new biomarker and potential therapeutic target for postoperative AKI in ATAAD. The results are preliminary and should be verified in other studies with larger sample sizes.

## Abbreviations

AKI, acute kidney injury; ATAAD, acute type A aortic dissection; TLR4, toll-like receptor 4; ICU, intensive care unit; RRT, renal replacement therapy; SCr, serum creatinine; ROC, receiver operating characteristic; OR, odds ratios; CI, confidence interval.
